# Rapid reviews may produce different results to systematic reviews: a meta-epidemiological study

**DOI:** 10.1016/j.jclinepi.2018.12.015

**Published:** 2019-05

**Authors:** Iain J. Marshall, Rachel Marshall, Byron C. Wallace, Jon Brassey, James Thomas

**Affiliations:** a School of Population Health and Environmental Sciences, King's College London, London, UK; b Independent Researcher, London, UK; c College of Computer and Information Science, Northeastern University, Boston, MA, USA; d TRIP Database, Newport, UK; e UCL Institute of Education, University College London, London, UK

**Keywords:** Rapid reviews, Systematic reviews, Meta-epidemiological studies, Research synthesis

## Abstract

**Objective:**

To simulate possible changes in systematic review results if rapid review methods were used.

**Study Design and Setting:**

We recalculated meta-analyses for binary primary outcomes in Cochrane systematic reviews, simulating rapid review methods. We simulated searching only PubMed, excluding older articles (5, 7, 10, 15, and 20 years before the search date), excluding smaller trials (<50, <100, and <200 participants), and using the largest trial only. We examined percentage changes in pooled odds ratios (ORs) (classed as no important change [<5%], small [<20%], moderate [<30%], or large [≥30%]), statistical significance, and biases observed using rapid methods.

**Results:**

Two thousand five hundred and twelve systematic reviews (16,088 studies) were included. Rapid methods resulted in the loss of all data in 3.7–44.7% of meta-analyses. Searching only PubMed had the smallest risk of changed ORs (19% [477/2,512] were small changes or greater; 10% [260/2,512] were moderate or greater). Changes in ORs varied substantially with each rapid review method; 8.4–21.3% were small, 1.9–8.8% were moderate, and 4.7–34.1% were large. Changes in statistical significance occurred in 6.5–38.6% of meta-analyses. Changes from significant to nonsignificant were most common (2.1–13.7% meta-analyses). We found no evidence of bias with any rapid review method.

**Conclusion:**

Searching PubMed only might be considered where a ∼10% risk of the primary outcome OR changing by >20% could be tolerated. This could be the case in scoping reviews, resource limitation, or where syntheses are needed urgently. Other situations, such as clinical guidelines and regulatory decisions, favor more comprehensive systematic review methods.

**What is new?**

**Key findings**
• Our simulation using data from the Cochrane Library found that rapid review methods may lead to different results than conventional systematic reviews.
• The degree of change to the pooled odds ratio of the meta-analysis varied substantially among the rapid review methods we examined.
• Searching only PubMed had the smallest risk of changed odds ratio. With this strategy 19% of meta-analyses had small changes or greater; 10% of meta-analyses had moderate changes or greater.

**What this adds to what was known?**
• Different methods for identifying studies in evidence synthesis have been widely examined, but mostly in the context of the effect on the number of studies found.
• More recently, researchers have started to examine whether missing studies has impact on the *results* of a review.
• This analysis is the largest to date which examines the effect of common rapid methods for study identification on meta-analysis results.

**What is the implication and what should change now?**
• PubMed-only searching might be considered in situations where a 10% risk of ≥20% change in odds ratio for the primary outcome is tolerable.
• This might be the case for scoping reviews, where there is resource limitation (financial or human), or where a synthesis is needed urgently.
• For uses demanding high accuracy (e.g., national guidelines and drug licensing decisions), a more comprehensive systematic review likely remains the best option.

## 1 Background

Systematic reviews are regarded as the gold standard method for evidence synthesis but are time-consuming and laborious to produce. Systematic reviews registered with PROSPERO take, on average, 67 weeks from protocol registration to publication [1].

This time frame can be too long, for example, in health emergencies, where decisions may need to be taken in weeks, days, or even hours, for example, during the 2014 Ebola epidemic [2], [3]. Systematic reviews are quickly outdated after publication [4] and resource limitation is an important reason why they are not kept up-to-date [5]. “Rapid” syntheses take methodological shortcuts and have become popular where syntheses are needed to tight deadlines, or where a conventional systematic review would be prohibitively expensive. Rapid syntheses have quickly become a fundament of national and international health policy, clinical guidelines, and health technology appraisals [3], [6], [7], [8], [9], [10], [11], [12].

A 2015 survey by Tricco et al. identified 50 unique rapid methods, which had been used in evidence synthesis; these often used less rigorous processes to identify studies to include [13]. Common rapid methods include limiting searches to a single database, limiting to English language, and limiting by publication date, among many others. By design these methods risk missing some studies but aim to produce results similar enough to those from more exhaustive systematic reviews to be useful. Wagner et al. conducted an online questionnaire of 556 guideline developers and policy makers, aiming to find what risk of error would be acceptable in rapid reviews [14]. They found that participants demanded very high levels of accuracy and would tolerate a median risk of a wrong answer^1^ of 10% (interquartile range [IQR] 5–15%). This was similar across public health, pharmaceutical treatment, and prevention topics.

Complex search strategies (e.g., combining database searching with hand-searching) retrieve more relevant studies compared with single database searches [15]. However, Egger et al. found that using mainstream bibliographic databases often produced similar statistical results in meta-analysis, as compared with more extensive searching [16]. Similarly, Hartling et al. found that removing non-English language studies and unpublished studies rarely affected the results of systematic reviews in pediatrics [17].

Glasziou et al. in their evaluation of 200 meta-analyses found that the results of the single “best” trial (defined as the study with the most precise effect estimate) agreed with the statistical significance of the associated meta-analysis in 81% of cases [18]. A reanalysis of 60 Cochrane systematic reviews examining different combinations of rapid search methods, found that conclusions were likely to change in 8–27% of cases, depending on the method, though the most reliable “rapid” method was still reasonably comprehensive (searches of MEDLINE, Embase, and CENTRAL plus manual searching of reference lists) [19]. A 2018 review compared 16 pairs of rapid and conventional systematic reviews (on the same topics). They found that both approaches yielded similar results for most reviews but identified two cases where the rapid review had important differences in conclusions [20]. To date, there has not been a large scale evaluation of the effects of rapid review methods on the numbers of lost studies and the resultant changes in meta-analysis results. This simulation study addresses this gap. Specifically, we evaluate the effects of strategies that can be implemented in early stages of the review (at the search and abstract screening stages). Enacting these strategies might reduce workload at these early stages and also later in the review process (see Box 1). Excluding studies early in the process could reduce the need for full-text retrieval, data extraction, and quality assessment; the latter tasks are commonly done in duplicate.

Box 1 **How work might be saved with the rapid review methods**

**Limiting search to PubMed only**
• Single search strategy to construct, and time saved searching (and learning how to use) multiple databases.
• No deduplication of citations from multiple databases required.
• Reduced search retrieval, hence fewer abstracts to screen.
• Fewer studies from which to extract data, quality assess, and input into meta-analysis.

**Limiting to a fixed search time period**
• Reduced search retrieval, hence fewer abstracts to screen.
• Fewer studies from which to extract data, quality assess, and input into meta-analysis.

**Setting a minimum number of participants**
• Easy rule for excluding studies at abstract screen stage (in principle abstracts could be excluded more quickly after quickly scanning for sample size).
• Where participant numbers are not reported in the abstract, the full text may still need to be scrutinized.
• Fewer papers to retrieve and studies from which to extract data, quality assess, and input into meta-analysis.

**Limiting to the largest trial only**
• Easily applicable rule for excluding studies, though all abstracts would still need to be scrutinized (to identify which trial is the largest).
• Fewer studies from which to extract data, quality assess, and input into meta-analysis.

## 2 Methods

### 2.1 Overview

**Fig. 1:**
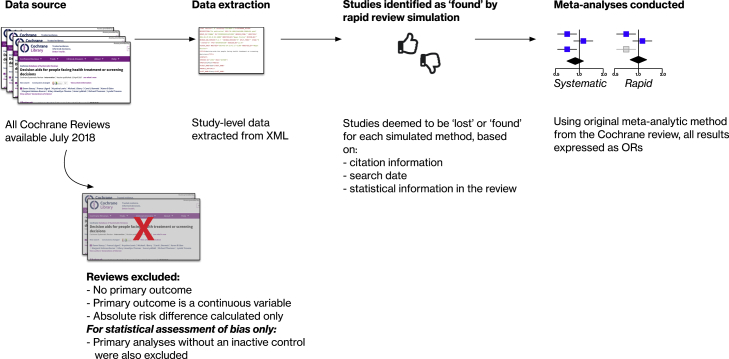
Overview of the study.

We conducted a meta-epidemiological study, simulating the effect of rapid review methods on meta-analyses in the Cochrane library. We reconducted all meta-analyses of binary primary outcomes, first including all studies (“systematic” method), and then repeating the meta-analysis after withholding studies we judged would be lost using a rapid review method (the “rapid” method). An overview of our approach is shown in Fig. 1. We focused on methods amenable to large-scale simulation using these data.

### 2.2 Eligibility criteria

We analyzed all systematic reviews from the Cochrane Database of Systematic Reviews (CDSR) published as of July 5th 2018 meeting the following criteria. Reviews were included if they included at least one meta-analysis, and if the primary outcome (numbered 1.1 in the reviews) was a binary variable and used a relative effect measure (risk ratio [RR] or odds ratio [OR]). All other systematic reviews were excluded. We chose binary outcomes in order that we could categorize changes in ORs.

### 2.3 Data extraction

All data extraction was scripted in Python from semistructured systematic review data (in XML format) made available to us for research purposes by the Cochrane Collaboration.^2^ We extracted the following data items: citation information for included studies, risk of bias assessments for included studies, primary outcome name and type (binary/continuous), meta-analysis method, event rates (number experiencing outcome and total number participants) from each arm of all included studies.

We conducted meta-analyses using the *meta* package in R, using the same methods as used in the original review (matching fixed/random effects models; Mantel-Haenszel/Peto/inverse variance methods)^3^ and expressed all as ORs [21]. At baseline, all original studies were included in the meta-analysis. We then simulated the effect of each rapid review method by withholding selected studies and redoing the meta-analysis.

We simulated the following rapid methods: searching PubMed only; limiting search by publication date (cutoffs of 5, 7, 10, 15, and 20 years before search date); limiting inclusion by sample size (minimum of 50, 100, and 200); and using the largest trial only. We chose these methods in particular, as they are commonly used in existing rapid syntheses [13] or proposed and evaluated in the evidence synthesis literature [18], [22]. We focused on methods that we could simulate algorithmically at scale. Other commonly used rapid methods (such as having abstracts screened by a single reviewer, limiting to English language only) are frequently described in the rapid review literature but were not amenable to a large-scale simulation. We therefore have not studied these methods exhaustively.

We describe how these methods would potentially reduce workload in Box 1.

### 2.4 Simulating PubMed-only searching

We did not reproduce the reviews’ PubMed search strategies, but instead assessed whether primary studies were indexed in PubMed. This strategy assumes that all studies indexed in PubMed would be findable.^4^ To determine which studies were PubMed indexed, we used three matching strategies. We first retrieved citation information for included studies from each systematic review. Article citations in systematic reviews frequently have minor typographical differences from their associated PubMed record. Therefore, a naive strategy of exactly matching article titles from the reference list to the PubMed entry would fail often. To overcome this, we made multiple searches of a local copy of the PubMed database for study citation fields from the original review (title, authors, publication date, and journal title), using a “fuzzy” matching algorithm for text strings^5^. Articles with a high degree of matching for all of these fields^6^ were deemed matches. We also searched Mendeley (using their search API^7^) and the Cochrane Register of Studies (using the unique Cochrane publication identifiers from the review) [23] to retrieve additional links to PubMed records. A statistical method known as *capture-recapture* is widely used in epidemiology to estimate the completeness of population-based disease registers, where the true prevalence of a disease might not be known [24]. We use the same method here to estimate the completeness of our set of linked PubMed records. Using this method, we estimate that 98.8% of linked PubMed records are retrieved by our multiple matching strategies. The full details of this analysis are presented in Appendix 1.

Studies are frequently described in multiple journal articles. Where this occurred, we used the reference to a primary publication (as marked in the original systematic review) and ignored any secondary publication records. Typically, the primary publication is the main journal article describing the key results of a trial, and secondary publications might include protocols, conference abstracts, or secondary analyses. In theory, considering the primary publication only might overestimate the difference between rapid and systematic syntheses. For example, consider a hypothetical meta-analysis, which included data on an outcome reported in a secondary publication. In this case, if the secondary publication happened to be indexed in PubMed, but the primary publication was not, we would have wrongly excluded the data in our analysis.

In practice, we assume this would happen rarely, particularly given that we are considering the primary outcomes in the systematic review only, which would be expected to be reported in the primary study publications.

To investigate what would happen if this assumption was not true, we conducted a sensitivity analysis, where we included studies if *any* of their publications were indexed in PubMed. The sensitivity analysis would be expected to underestimate degree of change in the rapid synthesis (because it optimistically assumes that all data relevant to the meta-analysis could be retrieved from the easiest-to-find publication) but would provide a lower bound on the likely effects of PubMed-only searching.

### 2.5 Limiting inclusion by publication date

To simulate time-limited searching, we retrieved the search date from the original systematic review. We then determined whether studies should be included based on the publication date taken from citation information in the systematic review.

### 2.6 Excluding smaller trials/largest trial-only strategies

For sample size cutoffs and largest trial strategies, sample sizes were estimated as the number of people from each study, who contributed data to the meta-analysis. This would comprise people who completed the study and had the outcome measured from the two arms forming the pairwise comparison of interest in the meta-analysis. This does not necessarily equal the number of participants randomized (e.g., for trials with >2 arms, or where not all participants randomized were included in the study analysis). For the largest trial-only strategy, odds ratios were calculated directly from the trial event rates.

### 2.7 Changes in pooled effect estimates

We initially defined effect size changes approximating Cohen's rule (where changes of ≥0.2 SDs are described as small, changes of ≥0.5 SDs as moderate, and ≥0.8 SDs as large). This system has been widely used for defining minimal important differences and is supported by empirical studies finding similar magnitude changes as useful cutoff points [25]. The SD of log ORs in our data set is 0.359, and hence this would have corresponded to 7%, 20%, and 33% relative change in the raw OR.^8^ We made a pragmatic decision to align our cutoffs with those used in other similar studies as far as possible [17], [26] and therefore rounded these figures, defining changes of pooled ORs of <5% as unimportant, <20% as small, <30% as moderate, and ≥30% as large. We defined “loss of all data” as cases in which the rapid method either did not find any trials or found only trials where the outcome does not occur in either arm contributing to the meta-analysis.

### 2.8 Changes in statistical significance

Statistical significance was calculated using the 95% confidence intervals of the meta-analysis result and were classed as changes from statistically significant to nonsignificant; from nonsignificant to significant; and a change in effect direction (i.e., a change from significance positive effect to negative effect or vice versa).

### 2.9 Bias due to rapid methods

We assessed both *risks* of bias (i.e., whether problems in the design and conduct of studies existed which risked biasing their results) and measured any systematic bias directly in the pooled effect estimates, comparing the results of the rapid vs. systematic syntheses. To assess risks of bias, we used the Cochrane Risk of Bias tool assessments of the original systematic review authors [27]. The types of bias assessed with this tool vary somewhat among reviews, and the systematic reviews did not use standardized text to describe the type of bias (e.g., problems in randomization are described as “Randomization method”, and “Adequate sequence generation?” among many others). We therefore manually mapped the free-text descriptions to the domains of the Cochrane tool. Because they were done most commonly, we evaluated risks of bias because of problems in random sequence generation, allocation concealment, blinding of participants and personnel, and blinding of outcome assessment.

The Cochrane Risk of Bias tool recommends that blinding should be assessed separately for each *outcome* in a study (e.g., inadequate blinding of trial participants may lead to a high risk of bias for subjective outcomes but is less likely to affect objective outcomes such as mortality, which could be rated as having a low risk of bias). Where a systematic review contained bias judgments for more than one outcome, we (manually) selected the judgment associated with the primary outcome.

To analyze the size and direction of any systematic bias because of the rapid methods, we used the subset of meta-analyses which had an inactive control (i.e., placebo, no treatment, or usual care arm; a complete definition is provided in Appendix 2). We reoriented all meta-analyses so that ORs <1 indicated a beneficial outcome with intervention. ORs >1 favored control. We calculated percentage changes in the pooled OR between systematic and rapid methods. The size and direction of systematic bias were assessed through visual inspection of histograms and calculating the mean percentage change in OR with 95% confidence intervals for the results from the rapid method vs. the results from the full systematic review.

## 3 Results

### 3.1 Data selection

**Fig. 2:**
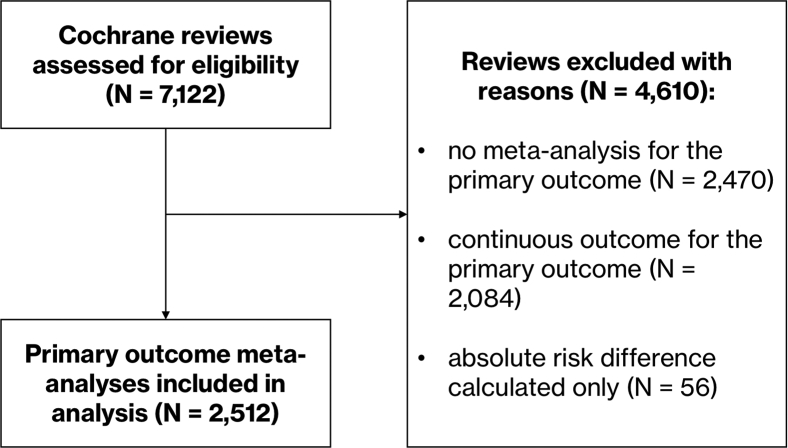
Flow diagram showing selection of meta-analyses for the analysis.

In July 2018, 7,122 systematic reviews were available in total in the Cochrane Database of Systematic Reviews. From these, we excluded 4,160 reviews as they lacked a suitable meta-analysis (no meta-analysis for a primary outcome = 2,470, continuous outcome assessed = 2,084, absolute risk difference calculated only = 56; see Fig. 2 for the flow of data).

The meta-analyses of the primary outcomes of the remaining 2,512 systematic reviews (including a total of 16,088 studies) were included in the final analysis. A description of the number of studies lost with each rapid method is presented in Table 1. Meta-analyses included a median of four studies (IQR 2–7).

**Table 1:** Effect of the rapid review methods on numbers of studies found and included in meta-analyses

Strategy	Total studies found (*N* = 16,088)	Median studies lost per meta-analysis (IQR) [baseline 4, IQR 2–7]
PubMed-only search (primary reference in PubMed)	14,255 (88.6%)	0 (0–1)
PubMed-only search (any reference in PubMed)^a^	14,540 (90.4%)	0 (0–1)
Search 5 yr	4,004 (24.9%)	2 (1–5)
Search 7 yr	5,437 (33.8%)	2 (1–5)
Search 10 yr	7,425 (46.2%)	2 (0–4)
Search 15 yr	10,225 (63.6%)	1 (0–2)
Search 20 yr	12,404 (77.1%)	0 (0–1)
Exclude <50 people	12,439 (77.3%)	1 (0–2)
Exclude <100 people	8,406 (52.3%)	2 (0–4)
Exclude <200 people	4,891 (30.4%)	2 (1–5)
Largest trial only	2,512 (15.6%)	3 (1–6)

a Sensitivity analysis.

### 3.2 Change in pooled effect estimates

**Fig. 3:**
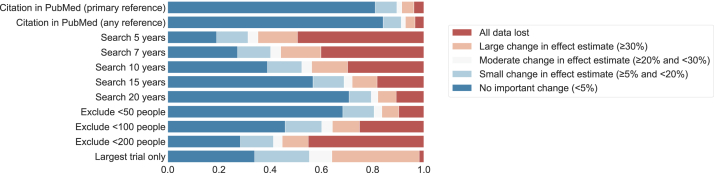
Change in pooled effect estimates with each rapid method.

Changes in the pooled effect estimates between the rapid and systematic methods are presented in Table 2 and Fig. 3. The proportion of meta-analyses with complete data loss varied widely between methods, with the largest trial-only strategy least likely to lose all data (<2% of cases)^9^ and limiting search to 5 years leading to greatest data loss (around half of cases).

**Table 2:** Changes in pooled effect estimates for rapid methods used (*n* = 2,512 meta-analyses)

Strategy	No important change	Small	Moderate	Large	All events lost	All studies lost
PubMed-only search (primary reference in PubMed)	2,035 (81.0%)	212 (8.4%)	48 (1.9%)	119 (4.7%)	4 (0.2%)	94 (3.7%)
PubMed-only search (any reference in PubMed)^a^	2,113 (84.1%)	177 (7.0%)	41 (1.6%)	94 (3.7%)	4 (0.2%)	83 (3.3%)
Search 5 yr	480 (19.1%)	307 (12.2%)	98 (3.9%)	387 (15.4%)	30 (1.2%)	1,210 (48.2%)
Search 7 yr	686 (27.3%)	323 (12.9%)	99 (3.9%)	394 (15.7%)	29 (1.2%)	981 (39.1%)
Search 10 yr	978 (38.9%)	337 (13.4%)	97 (3.9%)	352 (14.0%)	15 (0.6%)	733 (29.2%)
Search 15 yr	1,425 (56.7%)	304 (12.1%)	80 (3.2%)	244 (9.7%)	17 (0.7%)	442 (17.6%)
Search 20 yr	1,779 (70.8%)	217 (8.6%)	65 (2.6%)	179 (7.1%)	8 (0.3%)	264 (10.5%)
Exclude <50 people	1,717 (68.4%)	307 (12.2%)	76 (3.0%)	166 (6.6%)	5 (0.2%)	241 (9.6%)
Exclude <100 people	1,152 (45.9%)	358 (14.3%)	105 (4.2%)	265 (10.5%)	13 (0.5%)	619 (24.6%)
Exclude <200 people	713 (28.4%)	323 (12.9%)	87 (3.5%)	255 (10.2%)	10 (0.4%)	1,124 (44.7%)
Largest trial only	853 (34.0%)	536 (21.3%)	221 (8.8%)	856 (34.1%)	46 (1.8%)	0 (0%)

a Sensitivity analysis.

The strategy with the least impact on pooled effect estimates was simulated PubMed-only search (19% of results had ≥5% change), with the two next best strategies (limiting search to 20 years and excluding trials with <50 people), both leading to ≥5% change in ∼30% of meta-analyses. Limiting search to 5 years had greatest impact (82% of results with ≥5% change). A majority of effect size changes were small (8.4–21.3% of meta-analyses across the methods) but moderate (1.9–8.8%), and large changes (4.7–34.1%) were relatively common.

### 3.3 Change in statistical significance

**Fig. 4:**
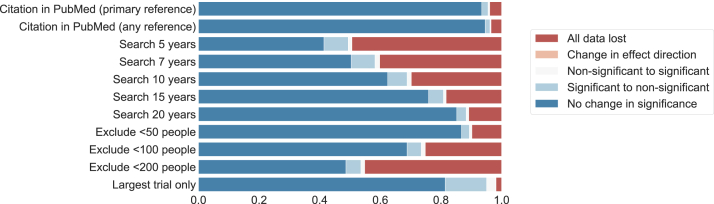
Change in statistical significance with each rapid method.

Changes in statistical significance with the rapid methods are presented in Table 3 and Fig. 4. The least impactful strategy was simulated PubMed-only search, which produced changes in statistical significance in 6.5% of cases. Excluding studies with <200 people led to changes in statistical significance in more than half of cases. With all methods, most changes were from statistically significant (in the systematic review) to nonsignificant with the rapid method, consistent with a loss of statistical power (2.1–13.7% of meta-analyses). “False positive” statistical significance (i.e., statistical significance in the rapid method, where the systematic review was nonsignificant) was relatively rare (0.6–3.0% of meta-analyses).

**Table 3:** Effect of rapid review methods on the statistical significance of results (*n* = 2,512 meta-analyses)

Strategy	No change in significance	Significant to nonsignificant	Nonsignificant to significant	Change in effect direction	All events lost	All studies lost
PubMed-only search (primary reference in PubMed)	2,348 (93.5%)	52 (2.1%)	14 (0.6%)	0 (0.0%)	4 (0.2%)	94 (3.7%)
PubMed-only search (any reference in PubMed)^a^	2,376 (94.6%)	40 (1.6%)	9 (0.4%)	0 (0.0%)	4 (0.2%)	83 (3.3%)
Search 5 yr	1,040 (41.4%)	201 (8.0%)	29 (1.2%)	2 (0.1%)	30 (1.2%)	1,210 (48.2%)
Search 7 yr	1,266 (50.4%)	197 (7.8%)	38 (1.5%)	1 (0.0%)	29 (1.2%)	981 (39.1%)
Search 10 yr	1,569 (62.5%)	160 (6.4%)	35 (1.4%)	0 (0.0%)	15 (0.6%)	733 (29.2%)
Search 15 yr	1,907 (75.9%)	123 (4.9%)	23 (0.9%)	0 (0.0%)	17 (0.7%)	442 (17.6%)
Search 20 yr	2,141 (85.2%)	78 (3.1%)	21 (0.8%)	0 (0.0%)	8 (0.3%)	264 (10.5%)
Exclude <50 people	2,180 (86.8%)	65 (2.6%)	21 (0.8%)	0 (0.0%)	5 (0.2%)	241 (9.6%)
Exclude <100 people	1,729 (68.8%)	117 (4.7%)	34 (1.4%)	0 (0.0%)	13 (0.5%)	619 (24.6%)
Exclude <200 people	1,222 (48.6%)	124 (4.9%)	32 (1.3%)	0 (0.0%)	10 (0.4%)	1,124 (44.7%)
Largest trial only	2,045 (81.4%)	343 (13.7%)	76 (3.0%)	2 (0.1%)	46 (1.8%)	0 (0.0%)

a Sensitivity analysis.

### 3.4 Bias with rapid methods

Differences in risks of bias are presented in Table 4. Studies “found” by the rapid methods were significantly more likely to have a low risk of bias than those “lost”. This applied to all rapid methods and for biases due to inadequacies in random sequence generation, allocation concealment, and blinding of outcome assessment. Studies missed by the rapid methods had an absolute risk increase of 8–25% of being at high risk of bias for these domains. The exception was in bias because of inadequate blinding of participants and personnel, where there was no difference in bias for the time-limited search strategies.

**Table 4:** Difference in risks of bias between studies “lost” and studies “found” by the rapid methods

Strategy	Random sequence generation (high or unclear risk of bias)			Allocation concealment (high or unclear risk of bias)			Blinding of participants and personnel (high or unclear risk of bias)			Blinding of outcome assessment (high or unclear risk of bias)		
	Studies lost	Studies found	*P*	Studies lost	Studies found	*P*	Studies lost	Studies found	*P*	Studies lost	Studies found	*P*
Citation in PubMed	862/1,328 (65%)	5,453/11,178 (49%)	<0.001	1,277/1,743 (73%)	7,665/13,665 (56%)	<0.001	511/708 (72%)	3,494/5,925 (59%)	<0.001	558/781 (71%)	3,707/6,608 (56%)	<0.001
Search 5 yr	5,285/9,307 (57%)	1,030/3,199 (32%)	<0.001	7,193/11,567 (62%)	1,749/3,841 (46%)	<0.001	2,948/4,859 (61%)	1,057/1,774 (60%)	0.44	3,248/5,398 (60%)	1,017/1,991 (51%)	<0.001
Search 7 yr	4,841/8,193 (59%)	1,474/4,313 (34%)	<0.001	6,486/10,196 (64%)	2,456/5,212 (47%)	<0.001	2,585/4,273 (60%)	1,420/2,360 (60%)	0.81	2,875/4,726 (61%)	1,390/2,663 (52%)	<0.001
Search 10 yr	4,129/6,625 (62%)	2,186/5,881 (37%)	<0.001	5,442/8,270 (66%)	3,500/7,138 (49%)	<0.001	2,125/3,516 (60%)	1,880/3,117 (60%)	0.94	2,404/3,896 (62%)	1,861/3,493 (53%)	<0.001
Search 15 yr	2,990/4,478 (67%)	3,325/8,028 (41%)	<0.001	3,831/5,564 (69%)	5,111/9,844 (52%)	<0.001	1,443/2,431 (59%)	2,562/4,202 (61%)	0.2	1,694/2,700 (63%)	2,571/4,689 (55%)	<0.001
Search 20 yr	1,977/2,819 (70%)	4,338/9,687 (45%)	<0.001	2,504/3,464 (72%)	6,438/11,944 (54%)	<0.001	962/1,560 (62%)	3,043/5,073 (60%)	0.25	1,132/1,734 (65%)	3,133/5,655 (55%)	<0.001
Exclude <50 people	1,760/2,738 (64%)	4,555/9,768 (47%)	<0.001	2,467/3,523 (70%)	6,475/11,885 (54%)	<0.001	967/1,488 (65%)	3,038/5,145 (59%)	<0.001	1,105/1,695 (65%)	3,160/5,694 (55%)	<0.001
Exclude <100 people	3,519/5,891 (60%)	2,796/6,615 (42%)	<0.001	5,001/7,409 (67%)	3,941/7,999 (49%)	<0.001	2,042/3,176 (64%)	1,963/3,457 (57%)	<0.001	2,255/3,533 (64%)	2,010/3,856 (52%)	<0.001
Exclude <200 people	4,844/8,676 (56%)	1,471/3,830 (38%)	<0.001	6,937/10,785 (64%)	2,005/4,623 (43%)	<0.001	2,966/4,667 (64%)	1,039/1,966 (53%)	<0.001	3,238/5,183 (62%)	1,027/2,206 (47%)	<0.001
Largest trial only	5,571/10,594 (53%)	744/1,912 (39%)	<0.001	7,781/12,978 (60%)	1,161/2,430 (48%)	<0.001	3,393/5,550 (61%)	612/1,083 (57%)	0.005	3,672/6,216 (59%)	593/1,173 (51%)	<0.001

*P* values calculated via chi-squared test.

The analysis of bias in the meta-analysis results is presented in Table 5, which shows the mean percentage changes in ORs with rapid method compared with systematic methods. Here, the meta-analysis outcomes were aligned so that a negative % change indicates that rapid methods favored the intervention arm on average; a positive % change indicates that rapid review methods favored control on average. A 0% change would indicate no systematic favoring of intervention or control with the rapid method. Table 5 shows that the mean percentage differences were small (i.e., indicating little or no systematic bias on average) and varied across different methods, from a −1.6% change (favoring intervention) with simulated PubMed-only searching to a +4.5% change (favoring control) with limiting search to 15 years. Most mean differences were not statistically significant, with the exception of 15-year search (+4.5%, 95% CI + 1.2% to +7.9%) and excluding samples of <200 people (+4.4%, 95% CI + 1.1% to +7.9%). Histograms showed that the changes in results appeared to have symmetrical distributions; these are presented in Appendix 2.

**Table 5:** Analysis of direction of change of meta-analysis results (excluding meta-analyses with no change); Negative % change is in favor of intervention efficacy

Strategy	Number of meta-analyses	% change (SD), 95% CI of mean
PubMed-only search (primary reference in PubMed)	1,497	−1.6 (35.3), −4.4 to +1.3
PubMed-only search (any reference in PubMed)^a^	1,503	−2.9 (29.4), −5.5 to −0.3
Search 5 yr	790	+3.5 (65.2), −0.6 to +7.8
Search 7 yr	924	+3.3 (71.2), −1.0 to +7.7
Search 10 yr	1,090	+1.8 (67.0), −2.1 to +5.8
Search 15 yr	1,262	+4.5 (47.5), +1.2 to +7.9
Search 20 yr	1,381	+2.5 (50.9), −1.3 to +6.4
Exclude <50 people	1,393	+1.9 (39.6), −0.8 to +4.7
Exclude <100 people	1,165	+2.5 (43.1), −0.1 to +5.3
Exclude <200 people	876	+4.4 (52.0), +1.1 to +7.9
Largest trial only	1,533	+3.3 (76.3), +0.0 to +6.7

a Sensitivity analysis.

### 3.5 Sensitivity analyses for PubMed-only searching

As previously described, the sensitivity analysis assumed that studies were “found” if *any* related article was present in PubMed (a somewhat optimistic assumption, but one which provides a useful lower bound on the PubMed search estimates). The sensitivity analysis found similar but modestly smaller impact of PubMed-only searching than our primary analysis for all outcomes. Overall, the sensitivity analysis found that PubMed-only search led to ≥5% change in pooled effect estimates in 15.9% of cases and changes of statistical significance in 5.4% of cases. The full breakdown of results is presented in Table 1, Table 2, Table 3. This similarity suggests our results are not likely to vary substantially even if data from secondary publications contributed to meta-analyses.

## 4 Discussion

Despite increasing reliance on rapid reviews to inform health policy and clinical practice, comparatively less had been known about the reliability of their results, and whether they met the standards decision makers require [14]. All the rapid methods assessed in our analysis led to small or greater changes exceeding the 10% tolerated “error rate” described in the survey by Wagner et al. [14]. For situations where a moderate change in odds ratio could be tolerated, PubMed-only search came very near to that acceptability threshold (10.6% risk of ≥20% change in results). Overall, the changes varied substantially in importance. Critically, not all changes we observe would be misleading. For example, a rapid review that finds no studies is not likely to lead to a false conclusion but could trigger a more comprehensive search (where resources allow). Similarly, a small change in effect size might often not lead to substantial changes in overall conclusions.

We assessed the effects of rapid review methods on meta-analyses results for a primary outcome only; we were not able to investigate rates of incorrect answers (as review conclusions do not typically depend on a single meta-analysis result but would be expected to depend on analysis from expert authors taking account of multiple beneficial and harmful outcomes and knowledge of the topic).

In practice, the decision concerning whether to use rapid review methods is complex; many factors other than an acceptable error rate are important. For example, where there are insufficient financial, human, or time resources, a full systematic review might be impossible. Rapid reviews with a higher risk of incorrect conclusion than those found acceptable by the participants in Wagner et al's. study might be preferable to no information at all [14]. Importantly, their assessment of acceptable error rates did not take into account the (substantial) opportunity cost of conducting comprehensive systematic review methods for selected topics, where reviews still do not exist for many clinically important questions.

Conversely, in a context where guideline authors may also have their processes subject to judicial review [28], [29] the results of an evidence synthesis not only need to be correct but also need to be seen to be correct. Failing to include potentially relevant research could undermine public confidence in the reliability of the process, even where the final result is not importantly affected.

We described in Box 1 the tasks avoided. However, there is comparatively little evidence about how much *time and effort* might be saved by rapid methods shortcuts. A survey of librarians found that translating a search strategy for additional databases took on average 5 hours, but with large variation, the maximum was 75 hours [30]. Shemilt et al. evaluated the cost-effectiveness of different abstract-screening workflows, including the use of two reviewers, one reviewer, and automation [31]. Such a design could prove useful in evaluating the time implications of other rapid review methods. In our analysis, since we did not directly reproduce the search (and hence have no knowledge of the volume of excluded articles), we could not infer time or labor saving from the methods examined.

A critical and unanswered question is whether methods shortcuts are directly responsible for faster turnaround, or whether other factors are at play. Rapid reviews are often commissioned, and therefore might be more likely to be undertaken by well resourced, experienced, and dedicated teams, with tightly tracked deadlines. In addition, rapid reviews are likely to be more focused in scope, which itself facilitates rapid production. How scope and working practices rather than methods affect the time taken to conduct a review remains poorly understood. Time saved by methods shortcuts also need to be examined in the context of the (typically large) total time and effort for a review. From the perspective of a single review, even if searching multiple databases took a little more time, it might not add meaningfully to a total period that could be as long as 2 years. However, where a large number of reviews are being done (e.g., national guideline organizations, Cochrane, and dedicated evidence synthesis groups), small performance improvements accrue and could become meaningful at scale.

Our analysis focuses on the primary meta-analysis from each review. Although this approach was chosen to identify the most important analyses, the balance of multiple outcomes may alter a review's conclusions. Similarly, for reviews examining adverse effects, information from smaller or more difficult-to-find studies might be important [32]. In systematic reviews that examine whether two interventions are equivalent (or similarly whether a new intervention is noninferior to an older one), the size of the confidence interval is critical [33]. We did not examine changes in confidence intervals in detail (other than as a binary “significant”/“nonsignificant”), and meta-analysis changes that we categorized as showing “no important difference” statistically could hide critical differences in this scenario.

### 4.1 Importance of results change

We assume that the methods used in the CDSR represent a gold standard. In practice, many reviews using a “full” systematic method do not adequately search for unpublished literature, and therefore themselves provide an imperfect estimate of treatment effects [34], [35], [36]. The most frequent error type in our analysis was loss of all data. Nearly half of all studies were lost overall when samples <100 were excluded. The meta-analyses in our source data incorporated a median of four studies, with studies having a median of 101 participants. Analyses with small numbers of studies with modest sample sizes will be particularly susceptible to change if some studies are not found.

It is possible that excluding small trials might improve the accuracy of the final result by reducing the possibility of small trials bias [37]. Dechartres et al. in their analysis of 93 meta-analyses found that studies with sample size <50 led to an exaggerated treatment effect of almost 50%, compared with trials having ≥1,000 participants [38]. Notably, only 18% of the meta-analyses analyzed here contained a study with ≥1,000 participants. Small trials have been found to be more heterogeneous [39] and susceptible to publication bias [37] than large trials; relying on the largest study alone might, in some cases, be more valid than a conventional systematic review approach. We additionally found that excluded studies were generally at higher risk of bias. If a rapid reviews strategy omitted smaller trials at higher risk of bias, changed conclusions might be more “correct” than the original.

This is similar to the findings of Egger et al., who investigated the effects of difficult-to-find studies (being those which are unpublished, non-English language, and not indexed in MEDLINE) via a secondary analysis of published meta-analyses [16]. They found that the difficult-to-find studies were less likely to have adequate allocation concealment or blinding and concluded that if resources were limited, researcher time might be better spent conducting a rigorous bias assessment of the articles produced from a limited literature search, rather than engaging in a comprehensive search. However, we did not find evidence of a systematic bias; most results from the rapid method were close to the full systematic review result, and deviations were equally likely to favor controls as interventions. We note that biases have been found to vary in their effect between studies and between different meta-analyses [40]. Our analysis therefore does not exclude the possibility of bias in some areas (e.g., a 0% mean change could mask a bias in rapid methods in favor of the intervention for one clinical specialty, where the bias operates in a different direction in trials in another specialty).

The analysis by Glasziou et al. found the trial with the most precise results (typically the largest study) agreed in statistical significance with full meta-analyses in 81% of cases [18]. This aligns (exactly) with the results presented here, where in 81% of cases the largest trial had the same statistical significance as the full meta-analysis. Our analysis adds that in 3% of cases, the largest trial was statistically significant, but the full meta-analysis was not, a potentially misleading result. In over a third of cases, we found a large difference in effect size between the largest trial and the full meta-analysis. Whether this strategy is useful would depend on the degree of approximation which would be acceptable for a particular use.

### 4.2 Strengths and weaknesses

This study provides the largest evaluation to date of common rapid review methods for study identification on a large corpus of systematic reviews, covering a wide range of clinical areas. We have been able to examine effects not only on the number of studies found but also on whether these change the results of the associated meta-analyses.

Our simulation closely approximated the rapid review methods as they would be used in practice but did have some areas of difference. We assumed the study population size was equal to the number of people who contributed to the meta-analysis (because these data were readily available). However, this number may not exactly match the number of people enrolled (typically reported in study abstracts), particularly if the study has additional arms that do not contribute data, or if there is a high dropout/withdrawal rate for the particular outcome assessed. Our strategy could be reproduced from the title and abstract by using the number of people in the arms of interest, rather than the total. Likewise, if our strategy for retrieving PubMed identifiers from included studies had imperfect recall, it would underestimate the study retrieval for searching PubMed only. We maximized recall by using three separate matching processes, estimated to achieve >98% recall via a capture-recapture analysis. Our simulation does assume that all studies with a PubMed record would have been identified by a PubMed search. It is possible that an imperfect search strategy in the original review missed a relevant study from PubMed, but that the study was identified by another means (e.g., search of additional databases, or hand searching). Our simulation would underestimate the impact of PubMed-only searching if this occurred. Indeed, a 2016 reanalysis of searches from 120 systematic reviews found that the *coverage* (being the percentage of relevant articles actually in the database) of MEDLINE (the major component of PubMed) was 92.3%. However, *recall* (being the percentage of relevant articles actually found using the systematic review's documented search strategy) was much lower at 72.6%.

We defined a single set of effect size cutoffs to characterize changes across many meta-analyses. Importantly, these describe the magnitude of change in statistical effect and not the clinical importance of that change for a population [25]. A very small change in effect size (below our selected threshold) might be important, for example, where the absolute risk of outcome is high (and hence a small relative difference with an intervention has greater impact) or with outcomes that are highly important to avoid (e.g., mortality). Synthesis producers should take these features into account when deciding on whether a rapid review shortcut might be justified.

Finally, we have examined the effects of a small selection of rapid review methods only and focused on the identification of studies. The effects of rapid review methods targeting other review processes (e.g., having a single reviewer screen abstracts or extract data, compared with the duplication of processes as done in conventional systematic reviews) are worthy of research. Likewise, the meta-analyses included in our study differed in their characteristics (in terms of clinical question, overall sample sizes, precision of pooled estimates, and risks of bias), and we did not examine analyses with continuous outcomes. Future research could investigate whether meta-analyses with certain characteristics are less liable to change when using rapid review methods compared with others.

## 5 Conclusions

Different rapid synthesis methods vary greatly in their impact, and the importance of changes to results is likely to vary substantially for difference use cases and topics. For researchers, the best performing strategy tested here (PubMed-only searching) might be considered in situations where odds ratio changes smaller than our moderate cutoff for the primary outcome could be tolerated (this strategy had a ∼10% risk of the OR changing by >20%). This might be the case for scoping reviews, where there is resource limitation (financial or human), or where a synthesis is needed urgently. For uses demanding high accuracy (e.g., national guidelines and drug licensing decisions), a more comprehensive approach likely remains the best option.

## Footnotes

Supplementary data related to this article can be found at https://doi.org/10.1016/j.jclinepi.2018.12.015.

## Footnotes

1 The magnitude of change which would lead an answer to be “wrong” was not precisely defined but left to the judgment of the participants.

2 These XML files are the output of Cochrane's RevMan software. Examples containing the systematic review statistical data may be downloaded from the webpage of any Cochrane review.

3 Statistical code is available at https://doi.org/10.5281/zenodo.1447087.

4 PubMed-indexed studies might not be found if a rapid review used an inadequate search query; this issue is considered further in the Discussion.

5 Fuzzy matching algorithms can detect closely matching but nonidentical text strings. In our case, this allowed matching references in Cochrane reviews even if there were minor differences from the PubMed record (because of, e.g., minor transcription errors or partial titles). We used the Python package *fuzzywuzzy* (https://github.com/seatgeek/fuzzywuzzy).

6 We define as “high degree” of match as 100% matching titles between Cochrane and PubMed records, except where the journal title, publication year, or first author also exactly matched, in which case an additional 3% discrepancy in titles was allowed, as measured by the fuzzy matching algorithm.

7 Mendeley is a reference management system, which allows public access to its extensive database of scholarly articles. We developed software to query its database using its API (or Application Programming Interface, https://dev.mendeley.com/), which allowed us to find PubMed links for thousands of articles.

8 *N*.B. that a fixed *absolute* change in the logarithm of an OR is associated with a constant *relative* change in the raw OR.

9 Noting that if the largest trial had no events in either arm, the meta-analysis would have no useful data.
