# Supplementary material for: Rapid reviews may produce different results to systematic reviews: a meta-epidemiological study
Source: J Clin Epidemiol. 2019 May;109:30–41. doi: 10.5281/zenodo.1447087 (PMC6524137; doi:10.5281/zenodo.1447087)
Supplement: Appendix 1 [file mmc1.docx]

Appendix 1

# Completeness of PubMed record matching

Our simulation of PubMed-only search depends on linking articles included in systematic reviews to PubMed records. In this Appendix we provide data on of the completeness of the linkage.

In the current analysis, we use data from 16,088 primary studies (being those included in the 2,512 meta-analyses we examine). To determine whether these studies would be findable in PubMed, we linked citation data in the original systematic review to an associated PubMed record (where such a record exists).

As part of a wider project, we sought PubMed links for 43,720 articles (of which articles describing the 16,088 primary studies in the main analysis are a subset). In the rest of the Appendix, we describe linkage adequacy of the 43,720 articles.

We use three sources for linking systematic review citations to PubMed records: Mendeley, the Cochrane Register of Studies (CRS), and fuzzy string matching to a local copy of PubMed. The technical linkage methods are described in the main article.

Studies are often described in multiple publications. For the primary analysis, we consider a study is indexed in PubMed if the *primary* reference in the systematic review has an associated PubMed record. For the sensitivity analysis, we consider a study to be indexed in PubMed if *any* of the associated references have a PubMed record.

Capture-recapture modeling, originally developed to estimate the size of animal populations, has become increasingly popular in epidemiological studies,[[1,2]](https://paperpile.com/c/Ebi1R9/a0xY+BiG9) software development, and census population estimates.[[3]](https://paperpile.com/c/Ebi1R9/ZDig) In the case of animal studies, animals are captured, marked, and released. This process is repeated on multiple *capture occasions*, allowing data to be built with the presence/absence of each known animal on each occasion. From this data, capture-recapture models are able to estimate how many animals have remained *unseen* and therefore give an estimate of the total population.

Here, we aim to ascertain how many studies in the corpus of meta-analyses are indexed in PubMed, given three (imperfect) strategies for obtaining the links. Each of our matching strategies (the CRS, Mendeley, and PubMed fuzzy matching) can be seen as capture occasions, which have the opportunity to ‘capture’ PubMed records. We use a log-linear capture-recapture model, as implemented in the *Rcapture* package for *R*,[[3]](https://paperpile.com/c/Ebi1R9/ZDig) which can estimate with 3 or more sources.[[1]](https://paperpile.com/c/Ebi1R9/a0xY)

Capture-recapture models assume a *closed population*: that is, the source population does not change between capture occasions.[[4]](https://paperpile.com/c/Ebi1R9/aYD5) Here, our data satisfies this requirement, since no studies are added or removed during the assessment (each matching strategy receives an identical list of studies).

Sources of variability can be accounted for in log-linear models, and specifically *temporal variability* (i.e. does the probability of capture vary between the matching strategies?), *unit heterogeneity* (does the probability of capture vary among the PubMed-indexed studies?), and *behavioural* *variability* (is the likelihood of capture independent in each sample?).

Here, we account for temporal variance since the strategies use different methods which might affect capture likelihood (via the ‘Mt’ model in the *Rcapture* package). Given we had identical structured fields for all the references, and an identical process was used for all studies within each matching strategy, we did not incorporate a unit heterogeneity effect.

Likewise, we assume that the capture likelihood is independent with each matching strategy (since each match strategy used independently derived data sources and separate methods). We therefore also did not incorporate a behavioural variability effect.

**ANALYSES OF MATCH QUALITY**

**Primary reference (main analysis)**

| Cochrane Register of Studies | Mendeley | PubMed fuzzy matching | **Matched using *any* strategy** |
| --- | --- | --- | --- |
| 23,743 | 31,723 | 31,949 | **38,108** |

Table 1. Number of studies matched to PubMed records (total studies = 43,720)


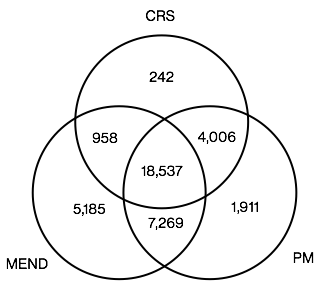


Figure 1. “Capture” of 38,108 studies with primary citation indexed in PubMed via the Cochrane Register of Studies (CRS), Mendeley (MEND), and fuzzy matching of PubMed records (PM)

Expected number of studies with PubMed records (via capture-recapture model):

38,558 (95% CI 38,514–38,603). Estimate of completeness of matching (i.e. observed number matches / expected): 98.8% (95% CI 98.7–98.9%)

**Any reference (sensitivity analysis)**

| Cochrane Register of Studies | Mendeley | PubMed fuzzy matching | **Matched using *any* strategy** |
| --- | --- | --- | --- |
| 29,752 | 32,623 | 33,300 | **43,720** |


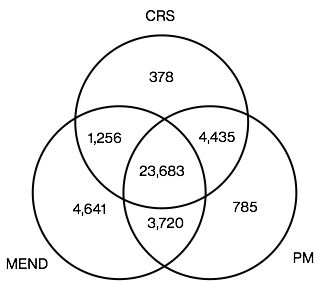


Figure 2. “Capture” of 43,720 studies with *any* publication indexed in PubMed via the Cochrane Register of Studies (CRS), Mendeley (MEND), and fuzzy matching of PubMed records (PM)

Expected number of studies with PubMed records (via capture-recapture model):

38,558 (95% CI 38,514–38,603). Estimate of completeness of matching (i.e. observed number matches / expected): 99.4% (95% CI 99.3–99.5%).

**PUBMED MATCHING CONSIDERATIONS**

We have developed our method of linking systematic review citation information with PubMed records iteratively over a number of years, and have used similar methods in previous studies.[[5,6]](https://paperpile.com/c/Ebi1R9/50hE+iuwd). This strategy aims for very high specificity (with the trade off of imperfect sensitivity, as described above) Accuracy was checked by manually validating samples of the output at each stage of development. In practice, 84% of articles were exact matches, and no fuzzy matching strategy was needed.

We occasionally found that an exchange of letters to the editor describing a study would arise as a false positive. Often the title would be identical, and in the case where the original authors had entered into correpondence the authors, year, and journal would also match.

However, in practice, the original study was also indexed in PubMed in every example we found of this nature. This is perhaps not surprising, given such correspondence nearly always takes place in the same journal. If PubMed indexes correspondence in that journal they will almost certainly index the research too. PubMed does not index conference proceedings in health journals, limiting another possible source of false matches of articles with near matching titles.

**REFERENCES**

[1] [Chao A, Tsay PK, Lin SH, Shau WY, Chao DY. The applications of capture-recapture models to epidemiological data. Stat Med 2001;20:3123–57.](http://paperpile.com/b/Ebi1R9/a0xY)

[2] [Tilling K, Sterne J a., Wolfe CD. Estimation of the incidence of stroke using a capture-recapture model including covariates. Int J Epidemiol 2001;30:1351–9; discussion 1359–60.](http://paperpile.com/b/Ebi1R9/BiG9)

[3] [Baillargeon S, Rivest L-P. Rcapture: Loglinear Models for Capture-Recapture in R. Journal of Statistical Software, Articles 2007;19:1–31.](http://paperpile.com/b/Ebi1R9/ZDig)

[4] [Capture-Recapture and Multiple-Record Systems Estimation I: History and Theoretical Development. Am J Epidemiol 1995;142:1047–58.](http://paperpile.com/b/Ebi1R9/aYD5)

[5] [Marshall IJ, Kuiper J, Wallace BC. Automating risk of bias assessment for clinical trials. Proceedings of the 5th ACM conference on Bioinformatics, computational biology, and health informatics, ACM; 2014, p. 88–95.](http://paperpile.com/b/Ebi1R9/50hE)

[6] [Marshall IJ, Kuiper J, Wallace BC. RobotReviewer: evaluation of a system for automatically assessing bias in clinical trials. J Am Med Inform Assoc 2016;23:193–201.](http://paperpile.com/b/Ebi1R9/iuwd)
