# Supplementary material for: Rapid reviews may produce different results to systematic reviews: a meta-epidemiological study
Source: J Clin Epidemiol. 2019 May;109:30–41. doi: 10.5281/zenodo.1447087 (PMC6524137; doi:10.5281/zenodo.1447087)

Appendix 2

# Assessing systematic biases in results

We used the following criteria to define which meta-analyses were eligible for inclusion in the assessment of systematic bias.

The following groups are counted as inactive controls:

- placebo
- sham or inactive treatments
- no treatment
- standard care (where interventions received by the control arm specifically described this as standard care, conventional care, or care as usual in the systematic review)
- active treatment received by both arms (i.e. A + B vs. B alone, where A vs no A is comparison of interest)
- waiting list arms

The following groups are excluded from the analysis:

- abmiguous direction of benefit (i.e. where the forest plot is not labeled with which treatment is favoured)
- both arms active comparitors
- old versus new treatments (except where explicitly described as standard care)
- trials of withdrawal of treatment, (i.e. stopping B, in a A+B vs B setup); subtly different from a control arm
- outcomes with ambiguous directionality (e.g. termination of pregnancy)

**DIRECTION OF CHANGE**

The following histograms show the *direction* of change for each rapid review method across all the meta-analyses. These analyses include the subset of meta-analyses with an inactive control (defined above). These plots correspond with the data described in Table 5 in the main manuscript.


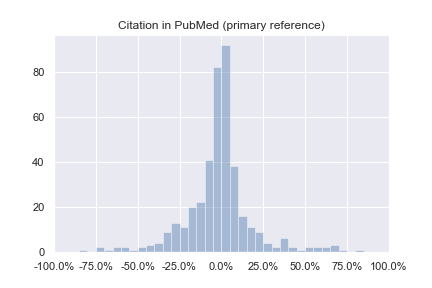


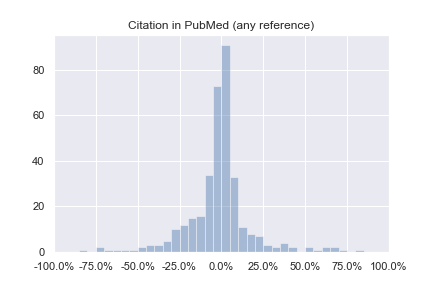


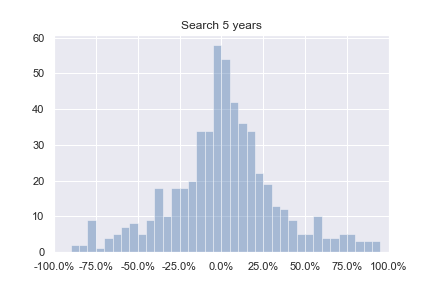


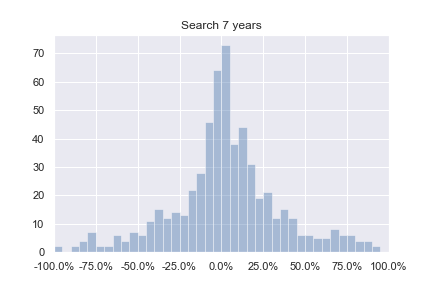


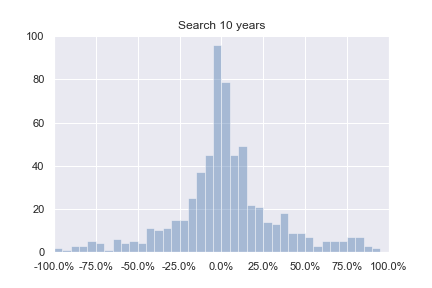


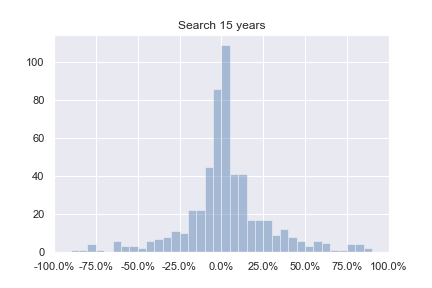


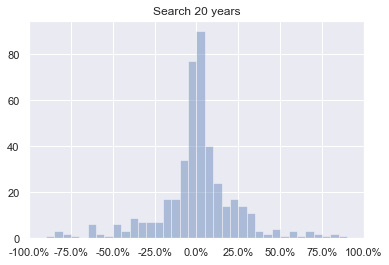


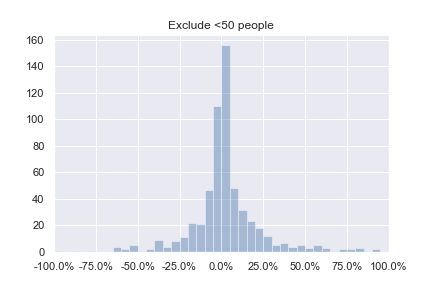


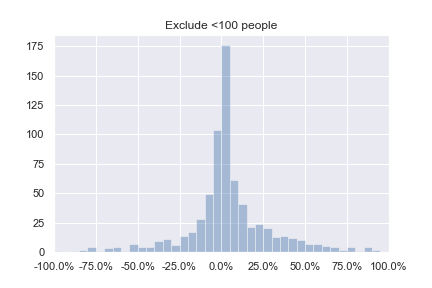


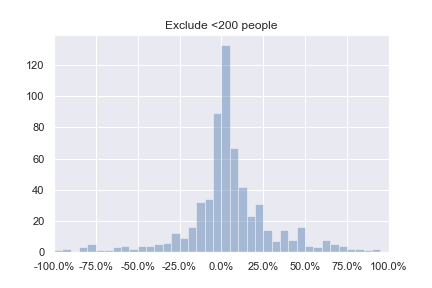


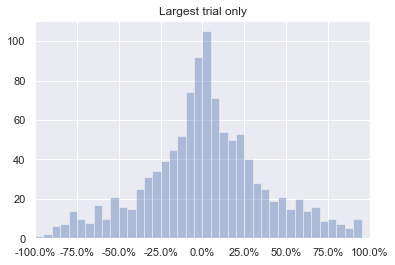

Supplement: Appendix 2 [file mmc2.docx]
